# Validity of traditional physical activity intensity calibration methods and the feasibility of self-paced walking and running on individualised calibration of physical activity intensity in children

**DOI:** 10.1038/s41598-020-67983-7

**Published:** 2020-07-03

**Authors:** Eero A. Haapala, Ying Gao, Anssi Vanhala, Timo Rantalainen, Taija Finni

**Affiliations:** 1 Biology of Physical Activity, Faculty of Sport and Health Sciences, University of Jyväskylä, PO Box 35, 40014 Jyväskylä, Finland; 2 Physiology, Institute of Biomedicine, School of Medicine, University of Eastern Finland, Kuopio, Finland; 3 Department of Sports Science, College of Education, Zhejiang University, Hangzhou, China; 4 Department of Education, Faculty of Educational Sciences, University of Helsinki, Helsinki, Finland

**Keywords:** Metabolism, Risk factors, Epidemiology, Paediatric research

## Abstract

There are no practical and valid methods for the assessment of individualised physical activity (PA) intensity in observational studies. Therefore, we investigated the validity of commonly used metabolic equivalent of tasks (METs) and pre-determined PA intensity classification methods against individualised PA intensity classification in 35 children 7–11-years-of-age. Then, we studied validity of mean amplitude deviation (MAD) measured by accelerometry during self-paced walking and running in assessment of individualised PA intensity. Individualised moderate PA (MPA) was defined as V̇O_2_ ≥ 40% of V̇O_2reserve_ and V̇O_2_ < ventilatory threshold (VT) and vigorous PA (VPA) as V̇O_2_ ≥ VT. We classified > 3–6 (or alternatively > 4–7) METs as MPA and > 6 (> 7) METs as VPA. Task intensities were classified according to previous calibration studies. MET-categories correctly identified 25.9–83.3% of light PA, 85.9–90.3% of MPA, and 56.7–82.2% of VPA. Task-specific categories correctly classified 53.7% of light PA, 90.6% of MPA, and 57.8% of VPA. MAD during self-paced walking discriminated MVPA from light PA (sensitivity = 67.4, specificity = 88.0) and MAD during self-paced running discriminated VPA from MPA (sensitivity = 78.8, specificity = 79.3). In conclusion, commonly used methods may misclassify PA intensity in children. MAD during self-paced running may provide a novel and practical method for determining individualised VPA intensity in children.

## Introduction

Free-living physical activity (PA) has been inversely associated with cardiometabolic risk in children^1^. Moreover, higher PA intensity may confer greater cardiometabolic health benefits than lower PA intensity^2–4^. Therefore, accurate assessment of PA intensity is important for informing health-related PA recommendations and in research on dose–response relationships. Nowadays, volume and intensity of PA in observational studies are typically assessed using accelerometers^5,6^. However, previous studies have used fixed acceleration magnitude cut-offs to define different PA intensities without accounting for individual variation in exercise capacity^6^. The continuing use of fixed cut-offs may have obscured our understanding on the prevalence of children accumulating recommended 60 min of daily moderate-to-vigorous PA (MVPA) and the role of PA intensity in health outcomes among youth^7,8^.

Fixed acceleration intensity cut-offs based on a single absolute acceleration magnitude value have been found to underestimate PA volume and intensity in lower fit and overweight adults^8^. Furthermore, metabolic equivalent of task (MET) is commonly used to assess PA intensity^9^ and to calibrate accelerometry cut-offs^10^. MET approach has been criticised because METs are confounded by body composition and METs also underestimate PA intensity in overweight and obese individuals^9^. In addition, previous calibration studies in children have provided several different cut-offs for light (LPA), moderate (MPA), and vigorous PA (VPA) leading to a large variation in the proportion of children meeting the PA recommendations^7,11,12^. However, some evidence suggests that of fixed acceleration magnitude cut-offs, those provided by Evenson et al.^13^ and Freedson et al.^14^ may be the most suitable for estimating PA intensity in children^15^. Nevertheless, these studies have subjectively decided calibration task intensities to determine LPA, MPA, and VPA irrespective of their true metabolic cost^7,11,12^. Therefore, previous studies do not have a strong physiological rationale for the suggested PA intensity cut-offs.

Expressing PA intensity relative to cardiorespiratory fitness (CRF) have been shown to reduce differences in PA volume and intensity between normal and overweigh adults and between adults with varying levels of CRF^8^. Therefore, some studies have recommended that PA intensity should be related to individualised oxygen uptake (V̇O_2_) reserve to account for the individual variation in CRF and the inability of fixed cut-offs to capture individualised PA intensity^16^. Therefore, defining PA intensity as a percentage of V̇O_2_ reserve provides more appropriate estimate of individualised PA intensity than fixed PA intensity cut-offs. However, because of individual variation in ventilatory threshold (VT), metabolic responses can differ significantly between individuals who exercise at the same proportion of their V̇O_2_ reserve^17,18^. PA performed below VT, a non-invasive equivalent of lactate threshold, has been considered as MPA that can be maintained prolonged periods without constantly increasing blood lactate concentration^19^. Furthermore, PA above VT leads to an increased lactate concentration and exhaustion and is often defined as VPA^19^. Exercise training intensity based on VT has also been shown to trigger larger physiological adaptations and to reduce individual variation in physiological responses to exercise compared to exercise training intensity based on percentage of V̇O_2_ reserve^20,21^ suggesting the validity of VT in the exercise prescription and calibration of VPA. Furthermore, some evidence in adults suggests that calibrating PA intensity cut-offs using lactate threshold or VT provides more accurate estimates of VPA than fixed cut-offs based on METs^22^. However, there are no previous studies utilising VT in calibration of accelerometry cut-offs for VPA in children.

Individual calibration of cut-offs may provide superior classification accuracy for LPA, MPA, and VPA in children^23^. Some studies have calibrated accelerometry cut-offs in a subsample of a larger study population and created fixed sample specific PA intensity cut-offs^24^ or used individually determined intensity cut-offs based on optimum walking speed and transition from walking to running during an incremental cardiopulmonary exercise test on a treadmill^25^. However, the individualised calibration of accelerometry cut-offs against true metabolic cost of activity or optimum walking speed and transition from walking to running during an incremental cardiopulmonary exercise test is not always feasible, because of their time consuming and resource intensive nature. On the other hand, self-selected walking speed has been found to reflect individualised MPA intensity^26^, and we hypothesised that self-preferred walking speed may be a feasible method to individually calibrate MPA. However, there are no studies on the validity of self-paced walking and running in the individual calibration of accelerometry cut-offs against V̇O_2_ reserve and VT in children.

To improve our understanding on the role of PA intensity it is essential to investigate the validity of commonly used methods used to define PA intensity and to investigate novel methods aiming to improve PA intensity estimation in children. Therefore, we first investigated the validity of PA intensity cut-offs based on METs and task-specific calibration activities against individualised PA intensity cut-offs in children. Second, we investigated whether acceleration mean amplitude deviation (MAD) normalised for MAD measured during self-paced walking and running provide better classification accuracy for individualised PA intensity than fixed MAD cut-offs.

## Results

### Characteristics of participants and different physical activities

**Figure 1:**
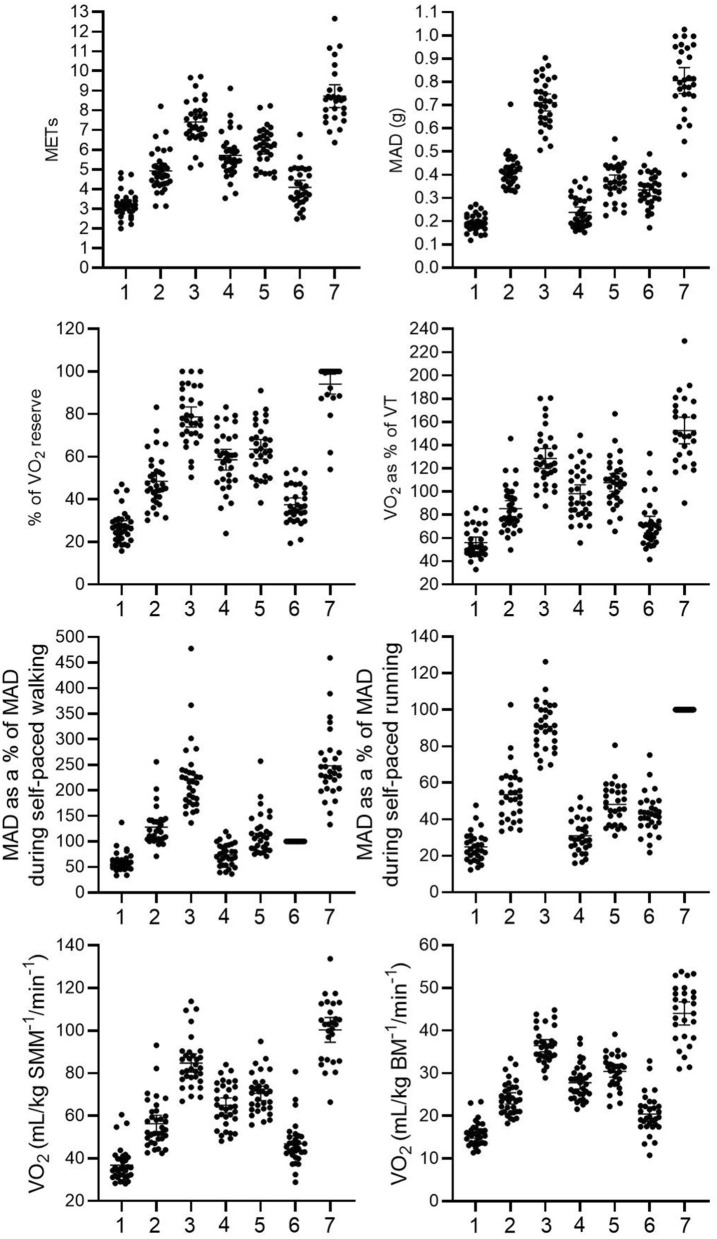
Differences in METs, MAD, V̇O_2_ as a % of V̇O_2_ reserve, V̇O_2_ as a % of V̇O_2_ at VT, and V̇O_2_ normalised for skeletal muscle mass (SMM) or body mass (BM) in different physical activities. 1: walking on a treadmill 4 km/h, 2: walking or running on a treadmill 6 km/h, 3: walking or running on a treadmill 8 km/h, 4: walking up and down the stairs, 5: playing hopscotch, 6: self-paced walking, 7: self-paced running. *MET* metabolic equivalent of task, *MAD* mean amplitude deviation, *V̇O*_*2*_ oxygen uptake, *VT* ventilatory threshold.

Characteristics of the participants are shown in Table 1. METs, absolute MAD, V̇O_2_ as a % of V̇O_2_ reserve, V̇O_2_ as a % of V̇O_2_ at VT, MAD relative to MAD during self-paced walking and running, and V̇O_2_ normalised for skeletal muscle mass (SMM) or body mass (BM) increased with increasing treadmill speed and were higher during self-paced running than during self-paced walking (p < 0.001 for main effect for all comparisons, Fig. 1). MAD did not differ between walking or running on a treadmill for 6 km/h and playing hopscotch (p = 0.126). METs, V̇O_2_ as a % of V̇O_2_ reserve, V̇O_2_ as a % of V̇O_2_ at VT, and V̇O_2_ normalised for SMM did no differ statistically significantly between walking up and down the stairs and playing hopscotch (p = 0.059 to 0.106).

**Table 1:** Characteristics of participants.

	**All**	**Girls**	**Boys**	**P**
Age (years)^a^	9.6 (3.0)	9.7 (2.9)	9.6 (2.8)	0.960
Stature (cm)	137.6 (9.2)	135.7 (9.3)	140.4 (8.7)	0.149
Weight (kg)	32.6 (6.9)	30.2 (6.0)	36.2 (6.8)	0.009
BMI (kg/m^2^)^a^	16.5 (3.6)	16.1 (2.2)	17.7 (4.8)	0.037
BMI-standard deviation score	− 0.2 (1.2)	− 0.5 (1.1)	0.3 (1.2)	0.052
Fat mass (kg)^a^	4.8 (5.0)	4.2 (4.2)	6.8 (7.5)	0.022
Body fat percentage (%)	16.6 (8.1)	15.7 (7.3)	18.0 (9.3)	0.433
Skeletal muscle mass (kg)	14.0 (2.9)	13.0 (2.5)	15.5 (2.8)	0.009
Maturity offset (years from estimated peak height velocity)	− 2.5 (1.2)	− 2.1 (1.2)	− 3.1 (0.9)	0.018
Resting V̇O_2_ (mL/min)^a^	153.9 (32.8)	146.4 (26.2)	189.3 (63.9)	0.006
Resting V̇O_2_ (mL/kg of SMM^−1^/min^−1^)	11.6 (1.7)	11.5 (1.6)	11.8 (1.8)	0.577
V̇O_2peak_ (mL/min^−1^) on a maximal cycle ergometer exercise test	1,333.1 (308)	1,224.5 (275.7)	1523.1 (275.6)	0.005
V̇O_2peak_ (mL/min) measured in all physical activities^b^	1,443.8 (300)	1,349 (235)	1619 (598)	0.016
V̇O_2peak_ (mL/kg of SMM^−1^/min^−1^) on a maximal cycle ergometer exercise test	95.8 (11.3)	95.0 (13.0)	97.3 (7.9)	0.569
V̇O_2_ at VT (mL/kg of SMM^−1^/min^−1^) on a maximal cycle ergometer exercise test	67.2 (9.8)	69.1 (11.7)	65.8 (5.6)	0.287
V̇O_2peak_ (mL/kg of SMM^−1^/min)^1^ measured in all physical activities^b^	103 (17.2)	98.4 (19.8)	103.8 (9.4)	0.576
V̇O_2_ at VT (% of V̇O_2peak_) on a maximal cycle ergometer exercise test	70.2 (6.9)	71.5 (7.4)	68.4 (5.8)	0.213
V̇O_2_ at VT (% of V̇O_2peak_) measured in all physical activities^b^	64.6 (10.2)	68.9 (13.9)	63.8 (7.8)	0.484

The data are mean (SD) or ^a^median (IQR).

^b^V̇O_2peak_ was defined as the highest V̇O_2peak_ during running on a treadmill for 8 km/h, self-paced running, or on a maximal cycle ergometer exercise test; *V̇O*_*2*_ oxygen uptake, *SMM* skeletal muscle mass.

### Validity of PA intensity based on MET-classification and task-specific classification against individualised PA intensity classification

**Figure 2:**
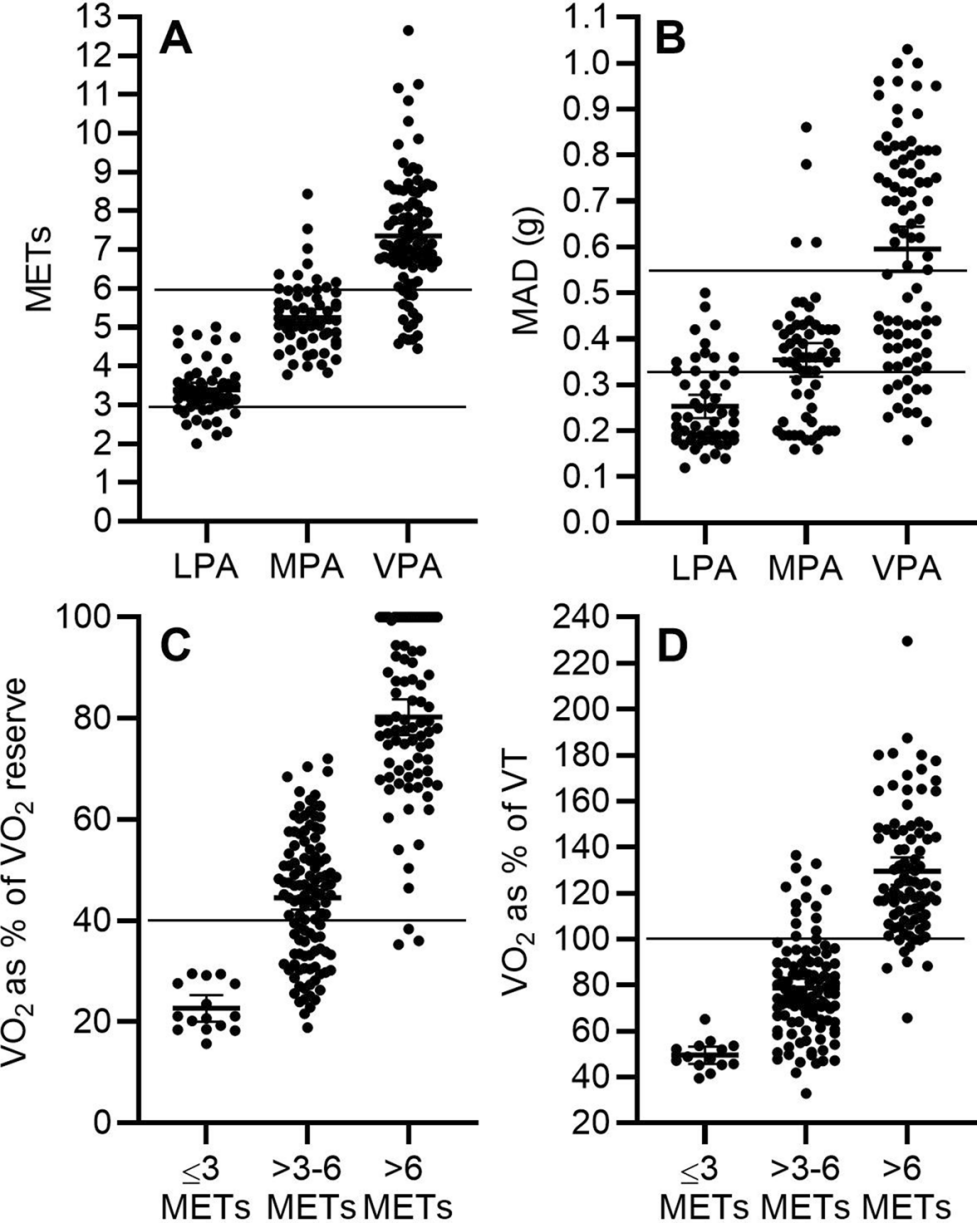
Agreement between individualised physical activity intensity categories based on V̇O_2_ reserve and ventilatory threshold and fixed physical activity intensity categories based on metabolic equivalent of tasks (METs) and mean amplitude deviations (MADs). (**A**) Individualised physical activity intensity versus METs. (**B**) Individualised physical activity intensity versus MADs, (**C**) MET-based physical activity intensity categories versus V̇O_2_ as % of V̇O_2_ reserve, (**D**) MET-based physical activity intensity categories versus V̇O_2_ as % of V̇O_2_ at ventilatory threshold. *LPA* individualised light physical activity (V̇O_2_ < 40% of V̇O_2_ reserve), *MPA* individualised moderate physical activity (V̇O_2_ ≥ 40% of VO_2_ reserve but < V̇O_2_ at VT), *VPA* individualised vigorous physical activity =  ≥ VO_2_ at VT). ≤ 3 METs = light physical activity, > 3–6 METs = moderate physical activity, > 6 METs = vigorous physical activity.

Compared to individualised PA intensity classification, MET-classification correctly classified 25.9% of LPA, 85.9% of MPA, and 82.2% of VPA (χ^2^(4) = 151, p < 0.001, Cramer’s V = 0.6, Fig. 2A,C,D). When we used > 4–7 METs to define MPA and > 7 METs to define VPA, 83.3% of LPA, 90.3% of MPA, and 56.7% of VPA (χ^2^(4) = 151, p < 0.001, Cramer’s V = 0.704) were correctly classified. Task-specific PA intensity classification correctly classified 53.7% of LPA, 90.6% of MPA, and 57.8% of VPA (χ^2^(4) = 147.3, p < 0.001, Cramer’s V = 0.595). Previously published fixed MAD cut-offs correctly classified 76.9% of LPA, 52.5% of MPA, and 54.4% of VPA (χ^2^(4) = 88.5, p < 0.001, Cramer’s V = 0.47, Fig. 2B).

### Physical activity intensity during self-paced walking and running

The mean of V̇O_2_ as a % of V̇O_2_ of reserve was 37.4% (min 19.3, max 54.1, SD = 8.7) and the median of V̇O_2_ as a % of V̇O_2_ at VT was 68.5% (min 41, max 133, IQR = 16.1) during self-paced walking (Fig. 1). Self-paced walking was categorised as LPA in 55.2% (N = 16), MPA in 34.5% (N = 10), and VPA in 10.3% (N = 3) of the children based on individualised PA intensity classification. Mean MET value during self-paced walking was 4.1 (min 2.5, max 6.8, SD = 1.0).

The median of V̇O_2_ as a % of V̇O_2_ on reserve was 100% (min 54.8, max 100, IQR = 10.9) and V̇O_2_ as a % of V̇O_2_ at VT was 152.5% (min 90.2, max 229.6, IQR = 29.1) during self-paced running. Self-paced running was MPA for 3.8% (N = 1) and VPA for 96.2% (N = 25) of the children. The median MET value during self-paced running was 8.5 (min 6.4, max 12.7, IQR = 1.4).

### MAD during self-paced walking and running in classifying individualised physical activity intensity

MAD as a % of MAD during self-paced walking was able to discriminate LPA from MVPA in 67.4% of the cases (Table 2). MAD as a % of MAD during self-paced running were able to discriminate VPA from MPA in 78.8% of the cases. Fixed MAD thresholds were able to discriminate LPA from MVPA in 65.8% of the cases and VPA from MPA in 66.7% of the cases. The ability to correctly identify individualised PA intensity increased when walking up and down the stairs and playing hopscotch were excluded from the data.

**Table 2:** Receiver operating characteristics curve analyses for the accuracy of individualised and fixed cut-offs to assess moderate and vigorous physical activity in children.

	**MAD relative to self-paced walking or running**					**Mean amplitude deviation (MAD)**				
	**AUC**	**Youden index**	**Cut-off (%)**	**Sensitivity**	**Specificity**	**AUC**	**Youden index**	**Cut-off (g)**	**Sensitivity**	**Specificity**
Individualised physical activity intensity classification										
MVPA^a^	0.828 (0.77 to 0.88)	0.554	100	67.4	88.0	0.842 (0.78 to 0.89)	0.561	0.37	65.8	90.4
VPA^b^	0.863 (0.81 to 0.91)	0.581	49.2	78.8	79.3	0.851 (0.79 to 0.90)	0.559	0.43	66.7	89.2
MVPA^a^^,^^c^	0.930 (0.87 to 0.97)	0.705	100	83.0	87.5	0.941 (0.89 to 0.97)	0.768	0.37	86.8	90.0
VPA^b,c^	0.945 (0.89 to 0.98)	0.823	63.8	91.1	91.3	0.957 (0.91 to 0.94)	0.830	0.50	87.7	95.2
MET-based physical activity intensity classification										
	AUC	Youden index	Cut-off (%)	Sensitivity	Specificity	AUC	Youden index	Cut-off (g)	Sensitivity	Specificity
> 3–6 METs (MVPA)^a^	0.806 (0.75 to 0.86)	0.558	100	55.8	100	0.858 (0.80 to 0.90)	0.648	0.33	64.8	100
> 6 METs (VPA)^b^	0.908 (0.86 to 0.94)	0.702	54.8	81.3	89.0	0.868 (0.82 to 0.91)	0.633	0.43	72.1	91.2
> 3–6 METs (MVPA)^a^^,^^c^	0.860 (0.79 to 0.91)	0.641	100	64.1	100	0.957 (0.91 to 0.94)	0.830	0.50	87.7	95.2
> 6 METs (VPA)^b,c^	0.988 (0.95 to 100	0.923	64.0	98.2	94.1	0.90 (0.84 to 0.94)	0.748	0.33	74.8	100
Task-specific physical activity intensity classification										
	AUC	Youden index	Cut-off (%)	Sensitivity	Specificity	AUC	Youden index	Cut-off (g)	Sensitivity	Specificity
MVPA^a^	0.930 (0.89 to 0.96)	0.778	85.3	84.2	93.6	0.935 (0.89 to 0.96)	0.831	0.27	83.1	100
VPA^b^	0.990 (0.97 to 1.00)	0.966	65.9	100	96.6	0.994 (0.97 to 1.00)	0.974	0.50	98.4	98.8
MVPA^a^^,^^c^	0.917 (0.86 to 0.96)	0.765	91.9	79.7	96.8	0.918 (0.86 to 0.96)	0.828	0.27	82.8	100
VPA^b,c^	0.988 (0.96 to 1.00)	0.967	65.9	100	96.7	0.994 (0.97 to 1.00)	0.974	0.50	98.4	99.0

^a^Self-paced walking as independent variable

^b^self-paced running as independent variable

^c^The data set excluding walking up and down the stairs and playing hopscotch

*MVPA* moderate to vigorous physical activity, *VPA* vigorous physical activity, *AUC* area under the curve, *MET* Metabolic equivalent of task. Within a predetermined task-specific intensity we classified walking on a treadmill for 4 km/h as LPA, running on treadmill for 6 km/h, walking up and down the stairs playing hopscotch, and walking around an indoor track on self-chosen speed as MPA, and running on a treadmill for 8 km/h and running around an indoor track on self-chosen speed as VPA according to previous calibration studies.

The ability of MAD as a % of MAD during self-paced walking and running and absolute MAD to discriminate PA intensities using different PA intensity classifications is presented in Table 2. Notably, MAD during self-paced walking and absolute MAD had higher sensitivity and specificity to correctly classify PA intensity based on task-specific calibration tasks compared to other methods used to classify PA intensity.

## Discussion

We found that defining PA intensity using fixed METs and task-specific classification methods may lead to large errors in the classification of individualised PA intensity in children. We also found that MAD values measured during different laboratory activities normalised to MAD values measured during self-paced running had acceptable sensitivity to discriminate individualised VPA from MPA. Furthermore, we showed that MAD values measured during different laboratory activities normalised to MAD during self-paced running was more accurate in discriminating VPA from MPA than absolute MAD especially when more complex and intermittent physical activities, such as walking up and down the stairs and playing hopscotch, were included in the analyses.

We observed that METs, task-specific calibration, or previous MAD cut-offs^27^ misclassified LPA in up to 70% and VPA in 20–45% of the cases. Furthermore, compared with 3 METs as a cut-off for MVPA and 6 METs as a cut-off for VPA, using > 4 METs and > 7 METs improved the classification accuracy to discriminate LPA from MVPA, but decreased the classification accuracy to differentiate VPA from MPA. These findings are in line with the observations in adults showing that fixed PA intensity cut-offs based on METs can lead to a significant misclassification of PA intensity and underestimate true intensity and volume of PA especially in overweight or unfit individuals^8^. To the best of our knowledge, similar findings have not been published in children. None of the previous studies utilising direct measurements of V̇O_2_ have anchored proposed PA intensity cut-offs on physiological thresholds based on V̇O_2_ data or took individual differences in V̇O_2peak_ into account^13,28,29^. Furthermore, we found only small difference in MAD-based fixed PA intensity cut-offs for MPA (0.062 g) and VPA (0.0583) between our study and the study by Aittasalo et al. using comparable task-specific calibration method^27^ suggesting that task-specific PA intensity calibration has relatively good agreement between samples, but the cut-offs do not accurately reflect true PA intensity. These findings together indicate that common methods used to classify PA intensity may cause remarkable bias in the assessment of individualised PA intensity in children.

We observed that MAD measured during different activities normalised for MAD measured during self-paced walking or running were able to discriminate MVPA and VPA, but the sensitivity of those measures to correctly classify PA intensity was lower than in previous studies^13,30^. Differences in the methods used to classify PA intensity most likely explain these differences. Most previous studies providing individualised^25^ or population specific^13,27,30,31^ fixed accelerometry cut-offs have used predetermined task-specific activities or evaluated the validity of the cut-offs using fixed MET-thresholds. We showed that the sensitivity and specificity of absolute MAD to discriminate task-specific PA intensity was above 80% but the ability of correctly classify individualised PA intensity was below 70%. Another reason for the discrepancy in classification accuracy with previous studies may be that our study included activities with low acceleration but high energetic demand, such as walking up and down the stairs which occurs frequently in free living, while physical activities in some previous studies have been simple ambulatory activities^30,31^. Therefore, our findings suggest that fixed accelerometry cut-offs have limited validity in the assessment of individualised PA intensity.

MAD relative to MAD during self-paced running had better sensitivity to discriminate VPA from LPA and MPA compared with fixed MAD cut-off. However, the classification accuracy of MAD during self-paced running and fixed MAD cut-offs was comparable when the data included only walking and running activities. Nevertheless, using only activities including walking and running in the calibration of PA intensity cut-offs may not reflect habitual physical activities in children and would lead to errors in the estimation of habitual PA intensity. All children, except one, operated at intensity above VT during self-paced running suggesting that anchoring VPA to MAD values measured during self-paced running reflects VPA in almost all children. Fixed MAD cut-off for VPA based on our sample and from previous samples^27^ underestimated PA intensity in 33–46% of the cases while cut-offs based on MAD as a % of MAD during self-paced running misclassified 21% of the cases. Therefore, our results suggest that MAD during self-paced running provides better estimate of VPA in children than fixed MAD cut-offs.

Against our hypotheses, our results do not support the superiority of MAD during self-paced walking in the individual calibration of MVPA in children. Relatively large variation between children in V̇O_2_ during self-paced walking varying from less than 20% up to 54% of V̇O_2_ reserve may partly explain our observation. Differences in neuromuscular maturation, which may have an effect on ability to control walking intensity and walking economy, may explain this large variation in V̇O_2_ during self-paced walking in children aged 7–11 years^32^.

The strengths of the present study include a valid and simultaneous assessment of V̇O_2_ and accelerometry during different activities, assessment of true resting V̇O_2_, V̇O_2peak_ and VT, and the use of individualised PA intensity in categorising LPA, MPA, and VPA. Our sample was also relatively representative to general Finnish population regarding V̇O_2peak_^33^. We also had variable physical activities mimicking normal daily activities performed by the children. It would have been optimal to include free play and tasks where participants would have been allowed to perform free tasks in their normal environments to increase the ecological validity of the study. V̇O_2peak_ and VT were assessed during a maximal cycle ergometer test and V̇O_2peak_ was adjusted using the data from the treadmill running or self-paced running if higher V̇O_2_ was observed during those activities. Therefore, it is possible that we have underestimated true V̇O_2max_ in some participants. A maximal treadmill exercise test with a supramaximal validation test would have needed to measure true V̇O_2max_ to optimally reflect ambulatory activities in the present study. Furthermore, we defined MVPA as V̇O_2_ ≥ 40% of V̇O_2_ reserve. Although several previous studies have also defined MVPA as 40–55% of V̇O_2peak_^5^, the physiological rationale for these limits to separate LPA from MVPA is lacking. Therefore, the classification accuracy of self-paced walking could have been different with another individulised intensity threshold. More research is warranted to fully understand PA intensity in children. Finally, although children were required to arrive at fasted state to the resting measurement, they were not asked to avoid all exercise a day before the resting measurements. Therefore, exercise during the previous day could have had some minor effect on the resting V̇O_2_ in the present study. However, children have been found to recover fast even from strenuous exercise^34^.

In conclusion, our results suggest that fixed MET-values or predetermined task-specific calibration activities should be used with caution in the classification of PA intensity or in the calibration of accelerometry cut-offs in children. Moreover, child-specific MAD values corresponding approximately 50% of the MAD values measured during self-paced running may provide more accurate, feasible, and practical method for individual calibration of VPA in children. Furthermore, our results suggest that fixed MAD cut-offs may be acceptable if children only walk and run, but not when they perform more complex and intermittent activities. Therefore, individualised MAD values may provide better estimates of VPA in real life setting. Further studies investigating the physiological responses to different exercise intensities and whether self-paced or optimum walking speed^25^, self-paced running, or the transition from walking to running can be used in individual calibration of PA intensity in large-scale observational studies in children are warranted. Finally, there is a need for further studies investigating how individualised PA volume and intensity are related to health and wellbeing in children.

## Methods

### Participants

This study was based on the laboratory phase of the Children’s Physical Activity Spectrum (CHIPASE) study^35^. A total of 35 children (21 girls, 14 boys) aged 7–11 years were recruited from local schools and volunteered to participate in the study. Children were included if they were apparently healthy and were able to perform the physical activities at moderate and vigorous intensities. Children with chronic conditions or disabilities were excluded from the study. The study protocol was approved by the Ethics Committee of the University of Jyväskylä. All children gave their assents and their parents/caregivers gave their written informed consents. The study was conducted in agreement with the Declaration of Helsinki.

### Study protocol

The participants visited the laboratory three times. At the first visit, research staff explained the research protocol to children and their parents. They were also familiarised to the laboratory environment and the measurement equipment. At the second visit, children arrived at the laboratory in the morning after 10–12 h overnight fast for the assessment of anthropometrics, body composition, and resting V̇O_2_. At the third visit, children were asked to perform following activities for 4.5 min in a random order interspersed with 1-min rest: sitting quietly, sitting while playing a mobile game, standing quietly, standing while playing a mobile game, playing hopscotch, walking up and down the stairs, and walking or running on a treadmill at 4, 6, and 8 km/h. They were also asked to walk and run around an indoor track at self-chosen speed for 4.5 min. At the end of the third visit, children performed maximal cardiopulmonary exercise test on a bicycle ergometer.

### Assessments

#### Body size and body composition

Stature was measured to the nearest 0.1 cm using a wall-mounted stadiometer. BM, SMM, fat mass, fat free mass, and body fat percent were measured by InBody 770 bioelectrical impedance device (Biospace Ltd., Seoul, Korea). Body mass index (BMI) was calculated by dividing body weight with body height squared and body mass index standard deviation score (BMI-SDS) was computed using the Finnish references^36^.

#### Oxygen uptake during rest and different physical activities

Mobile metabolic cart (Oxycon mobile, CareFusion Corp, USA) was calibrated and dead space was adjusted to 78 ml for the petite size of the face mask following the manufacturer’s recommendations. V̇O_2_, carbon dioxide production (V̇CO_2_) and respiratory exchange ratio (RER) were collected breath by breath and computed in non-overlapping 1 s epoch lengths. Resting V̇O_2_ was measured in the quiet room while a child lying down as still as possible watching an age-appropriate cartoons. Resting V̇O_2_ was determined as the mean value between the 15th and 25th minute of 30 min of supine rest when the steady state was reached^37^. When steady stated was not observed or there were abnormal spikes in the data between 15 and 25th minute, we visually determined the steady state from the whole measurement period for further analysis. The data were visually checked among six participants and the changes made in analysis window varied between 50 s and five minutes. In physical activities, V̇O_2_ was averaged over 2 min from the 3rd and 4th minutes of each task when the plateau in V̇O_2_ and V̇CO_2_ was observed^38^. Although the order of physical activities was randomised, we also confirmed that V̇O_2_ returned near to baseline levels during the 1-min rest (Supplementary figure 1).

#### Peak oxygen uptake and oxygen uptake at ventilatory threshold

Cardiorespiratory fitness was assessed by a maximal ramp exercise test on an electromagnetically braked Ergoselect 200 K electromagnetic cycle ergometer (Ergoline, Bitz, Germany)^33^. The protocol included 2-min resting period sitting on an ergometer, a 3-min warm-up with a workload of 20 W, and an incremental exercise period with increase of workload either by 1 W/3 s (totalling 20 W/minute for children > 150 cm), 1 W/4 s (totalling 15 W/minute for children 126–150 cm), or 1 W/6 s (totalling 10 W/minute for children ≤ 125 cm) until voluntary exhaustion^39^. The participants were asked to keep the cadence at 70–80 during the test. The test was terminated when the participant was unable to keep the cadence of 65 or required to stop. Participants were verbally encouraged to exercise until voluntary exhaustion.

Respiratory gas exchange was assessed directly by breadth-by-breadth method using the metabolic cart from the 2-min resting period sitting on the ergometer until the voluntary exhaustion and were averaged over 15-s periods. We defined peak cardiorespiratory capacity as the highest V̇O_2_ achieved in the exercise test (V̇O_2peak_) averaged over 15 s recorded during the last minute of the exercise test and normalised it for SMM. If higher V̇O_2_ was observed during running on a treadmill for 8 km/h or during self-paced running (N = 21) than during the maximal cycle exercise tests (N = 14), the higher V̇O_2_ value was used as a measure of V̇O_2peak_. Beat-by-beat heart rate (HR) was continuously recorded during the exercise test using Polar H7 HR sensor (Polar Electro, Kempele, Finland).

The cardiopulmonary exercise test was considered maximal if the primary and secondary objective and subjective criteria indicated maximal effort and maximal cardiorespiratory capacity (a plateau of V̇O_2_ regardless of increasing workload, HR > 85% of predicted, respiratory exchange ratio > 1.00, or flushing and sweating), and the exercise physiologist supervising the exercise test considered the test maximal^40^.

V̇O_2_ at VT was individually determined by two exercise physiologists using modified V-slope method^41^ and any disagreements were solved by these two exercise physiologists. The VT was identified as a time point where the increase in V̇CO_2_ was steeper than the increase in V̇O_2_ during the maximal cardiopulmonary exercise test on a cycle ergometer. In determination of VT, we used data averaged over 15 seconds^41^. V̇O_2_ at VT was verified utilising the equivalents for V̇_E_/V̇CO_2_ and V̇_E_/V̇O_2_. According to equivalent method V̇O_2_ at VT was defined as a rate of V̇O_2_ where V̇_E_/V̇O_2_ begins to increase without an increase in V̇_E_/V̇CO_2_.

#### Accelerometry during different physical activities

Movement was measured by triaxial accelerometer (X6-1a, Gulf Coast Data Concepts Inc., Waveland, USA). We used raw acceleration data in actual g-units with the high range up to 6 g with 16-bit A/D conversion, and sampling at 40 Hz. The resultant acceleration of the triaxial accelerometer signal was calculated from $\sqrt{{x}^{2}+{y}^{2}+{z}^{2}}$, where x, y and z were the measurement sample of the raw acceleration signal in x-, y-, and z-directions. The X6-1a accelerometer has been shown to produce congruent results with the ActiGraph GT3X accelerometer in children^42^. The MAD was calculated from the resultant acceleration in non-overlapping 1 s epoch. MAD describes the mean distance of data points around the mean ( $\frac{1}{n}\sum_{i=1}^{n}|{r}_{i -}\stackrel{-}{r}|$ where n was the number of samples in the epoch, ${r}_{i}$ is the *i*th resultant sample within the epoch and $\stackrel{-}{r}$ is the mean resultant value of the epoch)^27,43^. The mean of the 1 s MAD values (g) were calculated in the 2 min time epochs for each activity and in 10 min epoch for lying down and were reported as the outcomes. MAD measured during different activities as a percentage of MAD measured during self-paced walking or running was computed as (MAD_activity_/MAD_self-paced walking or running_) * 100. To obtain individualised child-specific MAD values representing the cut-offs for MPA and VPA, the absolute child-specific MAD value measured during self-paced walking or self-paced running should be used and the appropriate multiplier provided in the Table 2 should be applied.

### Definitions of intensity in different physical activities

*Individualised intensity classification*. We defined individualised PA intensity using V̇O_2_ reserve and V̇O_2_ at VT. V̇O_2_ reserve was calculated as (V̇O_2_ during PA task/V̇O_2peak_—V̇O_2_ during rest) × 100. LPA was defined as V̇O_2_ below 40% and MPA as V̇O_2_ ≥ 40% of V̇O_2_ reserve^16^ to < V̇O_2_ at VT^19^. VPA was defined as V̇O_2_ at or above V̇O_2_ at VT^19^. MVPA was defined as activity V̇O_2_ ≥ 40% of V̇O_2_ reserve^16^.

*MET-based intensity classification*. MET values were computed as V̇O_2_ measured during the physical activities/V̇O_2_ during supine rest. PA intensity during different tasks was categorised as follows: LPA was defined as > 1.5–3 METs, MPA as > 3–6 METs, and VPA as > 6 METs^3^. We also performed additional analyses using the cut-offs of > 4 METs for MPA and > 7 METs for VPA. MVPA was defined as > 3 or > 4 METs.

*Predetermined task-specific intensity classification*. We classified walking on a treadmill for 4 km/h as LPA, running on treadmill for 6 km/h, walking up and down the stairs playing hopscotch, and walking around an indoor track on self-chosen speed as MPA, and running on a treadmill for 8 km/h and running around an indoor track on self-chosen speed as VPA according to previous calibration studies^7,11,12^. MVPA was then classified as tasks excluding tasks considered LPA. We also investigated the agreement of previously published fixed MAD cut-offs for LPA (< 332 mg), MPA (332 mg), and VPA (558.3 mg) based on predetermined task-specific intensity classification provided by Aittasalo et al.^27^ with individualised PA intensity.

### Statistical methods

Basic characteristics between girls and boys were compared using Student’s t-test for normally distributed continuous variables and Mann–Whitney U-test for skewed continuous variables. We investigated differences in METs, absolute MAD, V̇O_2_ as a % of V̇O_2_ reserve, V̇O_2_ as a % of V̇O_2_ at VT, MAD relative to MAD during self-paced walking and running, and V̇O_2_ normalised for SMM or BM in different physical activities using mixed-effects repeated measures ANOVA. We investigated the validity of MET-based PA intensity classification, task-specific intensity classification, and previously published fixed MAD^27^ cut-offs against individualised PA intensity cut-offs using χ^2^ test and Cramer’s V.

We used receiver operating characteristics (ROC) curves to investigate the optimal cut-off for MAD as a % of MAD during self-paced walking or running and absolute MAD to differentiate LPA from MVPA and LPA and MPA from VPA using individualised PA intensity classification. The area under the curve (AUC) is considered a measure of the effectiveness of the predictor variable to correctly discriminate MVPA from LPA and VPA from MPA (sensitivity) and to correctly discriminate LPA from MVPA and MPA from VPA (specificity). An AUC of 1 represents the ability to perfectly identify MVPA or VPA from other intensities, whereas an AUC of 0.5 indicates no greater predictive ability than chance alone. The optimal cutoff was determined by the Youden index^44^, which is the maximum value of *J* that is computed as: sensitivity + specificity − 1.

Student´s t-test, the Mann–Whitney U-test, and the χ^2^ test were performed using the SPSS Statistics, Version 23.0 (IBM Corp., Armonk, NY, USA). The data were visualised and the mixed-effects repeated measures ANOVA were performed by the GraphPad Prism, version 8.0.2 (Graph Pad Software, Inc., San Diego, CA, USA). The ROC curve analyses were performed using MedCalc Statistical Software, Version 16.1 (MedCalc Software bvba, Ostend, Belgium).

## Supplementary information

Supplementary figure 1

Supplementary information

## Data Availability

The datasets generated during and/or analysed during the current study are available from the corresponding author on reasonable request.
