# Supplementary figures and images for: Validity of traditional physical activity intensity calibration methods and the feasibility of self-paced walking and running on individualised calibration of physical activity intensity in children
Source: Sci Rep. 2020 Jul 3;10:11031. doi: 10.1038/s41598-020-67983-7 (PMC7335054; doi:10.1038/s41598-020-67983-7)

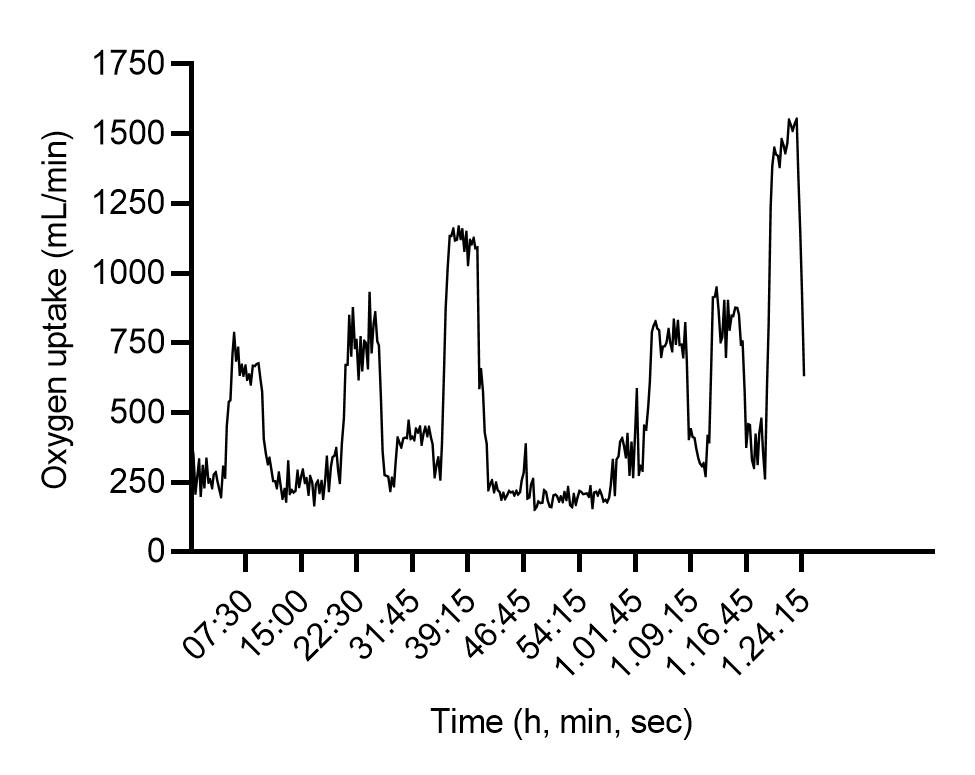

Supplement: Supplementary file 1 — Supplementary figure 1 [file 41598_2020_67983_MOESM1_ESM.jpg]
