# Supplementary material for: Validity of traditional physical activity intensity calibration methods and the feasibility of self-paced walking and running on individualised calibration of physical activity intensity in children
Source: Sci Rep. 2020 Jul 3;10:11031. doi: 10.1038/s41598-020-67983-7 (PMC7335054; doi:10.1038/s41598-020-67983-7)
Supplement: Supplementary file 2 — Supplementary information [file 41598_2020_67983_MOESM2_ESM.docx]

**Validity of traditional physical activity intensity calibration methods and the feasibility of self-paced walking and running on individualised calibration of physical activity intensity in children**

Eero A. Haapala^1,2^, Ying Gao^1,3^, Anssi Vanhala^1,4^, Timo Rantalainen^1^, Taija Finni^1^

*^1^Faculty of Sport and Health Sciences, University of Jyväskylä, Jyväskylä Finland; ^2^Physiology, Institute of Biomedicine, School of Medicine, University of Eastern Finland, Kuopio, Finland*

*^3^Department of Sports Science, College of Education, Zhejiang University, Hangzhou, China.*

*^4^Department of Education, Faculty of Educational Sciences, University of Helsinki, Helsinki, Finland*

**Figure caption for supplementary figure**

An example of raw oxygen uptake (V̇O_2_) data during different tasks.
